# Derivation of Land Surface Temperature for Landsat-8 TIRS Using a Split Window Algorithm

**DOI:** 10.3390/s140405768

**Published:** 2014-03-25

**Authors:** Offer Rozenstein, Zhihao Qin, Yevgeny Derimian, Arnon Karnieli

**Affiliations:** 1 The Remote Sensing Laboratory, Jacob Blaustein Institutes for Desert Research, Ben-Gurion University of the Negev, Sede Boker Campus, Midreshet Ben-Gurion 84990, Israel; E-Mail: oferroz@yahoo.com; 2 Institute of Agricultural Resources and Regional Planning, Chinese Academy of Agricultural Sciences, Beijing 100081, China; E-Mail: qinzh@caas.net.cn; 3 Laboratoire d'Optique Atmosphérique, Université de Lille1/CNRS, Villeneuve d'Ascq 59655, France; E-Mail: Yevgeny.Derimian@univ-lille1.fr

**Keywords:** thermal remote sensing, TIRS, Landsat-8, land surface temperature

## Abstract

Land surface temperature (LST) is one of the most important variables measured by satellite remote sensing. Public domain data are available from the newly operational Landsat-8 Thermal Infrared Sensor (TIRS). This paper presents an adjustment of the split window algorithm (SWA) for TIRS that uses atmospheric transmittance and land surface emissivity (LSE) as inputs. Various alternatives for estimating these SWA inputs are reviewed, and a sensitivity analysis of the SWA to misestimating the input parameters is performed. The accuracy of the current development was assessed using simulated Modtran data. The root mean square error (RMSE) of the simulated LST was calculated as 0.93 °C. This SWA development is leading to progress in the determination of LST by Landsat-8 TIRS.

## 1. Introduction

Land surface temperature (LST) is related to surface energy and water balance, at local through global scales, with principal significance for a wide variety of applications, such as climate change, urban climate, the hydrological cycle, and vegetation monitoring [1–4]. LST variations in space and time, measured by satellite remote sensing, are used for the estimation of a multitude of geophysical variables, such as evapotranspiration, vegetation water stress, soil moisture, and thermal inertia [5–7]. With the increasing recognition of the importance of LST, methods for its estimation from space have continuously been developed [8]. In recent decades, sensors, such as the Moderate-resolution Imaging Spectroradiometer (MODIS) and the Advanced Very High Resolution Radiometer (AVHRR), have provided public domain global thermal data twice daily, using two long-wave infrared (LWIR) bands. Landsat-5 Thematic Mapper (TM) and Landsat-7 Enhanced Thematic Mapper Plus (ETM+) provide thermal data using just one long-wave infrared (LWIR) band, with a higher spatial resolution but with a 16-day temporal resolution. Since satellite remote sensing provides a repetitive synoptic view in short intervals of the Earth's surface, it is a vital tool for monitoring LST.

**Figure 1.**
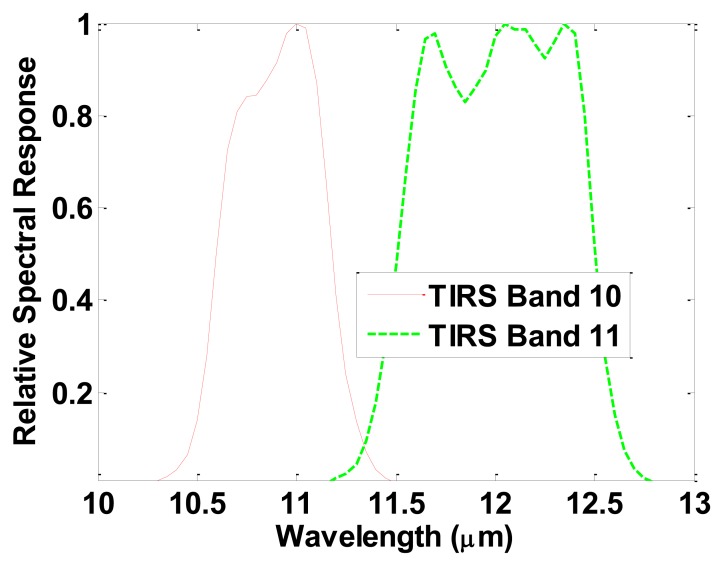
Landsat-8 TIRS bands' relative spectral response functions (the data can be seen in [16]).

Landsat-8 was successfully launched on 11 February 2013 and deployed into orbit with two instruments on-board: (1) the Operational Land Imager (OLI) with nine spectral bands in the visual (VIS), near infrared (NIR), and the shortwave infrared (SWIR) spectral regions; and (2) the Thermal Infrared Sensor (TIRS) with two spectral bands in the LWIR. The relative spectral response of the TIRS bands is presented in Figure 1. The two TIRS bands were selected to enable the atmospheric correction of the thermal data using a split window algorithm (SWA) [9,10]. The use of two separate, relatively narrow, thermal bands has been shown to minimize the error in the retrieval of LST [11]. The spatial resolution of TIRS data is 100 m with a revisit time of 16 days, and as a result, applications are different than those of other sensors with coarser spatial resolutions and shorter revisit times. While Landsat-8 images are already freely distributed through the U.S. Geological Survey (USGS), to the best of our knowledge, no SWA for LST retrieval from TIRS has been published. Although several SWAs have been developed for use with other sensors [12–15], some adaptations are required in order to implement them for the TIRS spectral bands. Therefore, the objective of this letter is to develop a SWA, adapted for use with Landsat-8 TIRS data, along with its accuracy assessment.

## 2. Split Window Algorithm (SWA)

The SWA was first proposed by McMillin [17] who suggested using the differences in the atmospheric absorbance of two adjacent LWIR bands in order to accurately retrieve the sea surface temperature (SST). In order to make the transition from SST to LST retrieval, one has to assume the land surface emissivity (LSE) in both bands *a priori* [12]. Qin *et al.* [18] have presented a SWA for AVHRR that requires only two essential variables: LSE and atmospheric transmittance. They tested their algorithm and found its accuracy to be 1.75 °C for real world data. Additionally, they found that it was preferable to the other SWAs that also performed well but required some parameters that are difficult to estimate [19,20]. Therefore, the SWA suggested by Qin *et al.* [18] was chosen to be adapted for TIRS, not only because it was tested and proved to be accurate, but also because it is reasonable to estimate its input parameters, as will be discussed in the next section of the paper. Further, the current work complements that of Qin *et al.* [18] to which we refer the reader for theoretical background and algorithm development.

The adaptation of the SWA for TIRS bands relies on the determination of parameter *L_i_* for the TIRS-specific spectral bands. *L_i_* is defined as:

(1) Li=Bi(T) /[∂Bi(T)/∂T]

in which *L_i_* has the dimension of temperature in Kelvin, *B_i_*(*T*) is the Planck function radiance, spectrally integrated over each of the TIRS bands at temperature *T*, and ∂*B_i_*(*T*) is the derivative of the Planck function for band *i* at temperature *T*. Therefore, ∂*B_i_*(*T*)/∂*T* can be calculated as:

(2) ∂Bi(T)/∂T≈[Bi(T+ΔT)−Bi(T)]/ΔT

**Figure 2.**
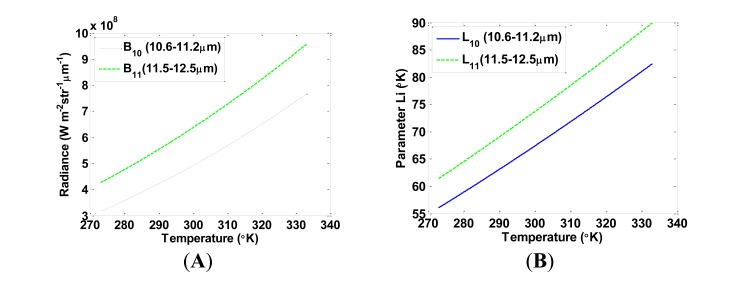
Variations of (**A**) Planck's radiance and (**B**) *L_i_* parameter with temperature for each of the TIRS bands.

We computed *L_i_* numerically by using ∂*B_i_*(*T*)/∂*T* and accurately fit it using a linear regression *L_i_* = *a_i_* + *b_i_T*. For the temperature range of 0–60 °C, *L_10_* = −64.4661 + 0.4398*T* (*r^2^* = 0.9968, standard error of estimation (SEE) = 0.1643) and *L_11_* = −68.8678 + 0.4755*T* (*r*^2^_=0.9967_, SEE = 0.1687) (Figure 2). However, coefficients *a_i_* and *b_i_* vary when computed for different temperature ranges, as presented in Table 1. From this table, we can see that as the range of *T* decreases, a better accuracy, or a lower SEE, is achieved. In this paper, the SWA accuracy assessment was carried out for the strict case of the extreme temperature range of 0–60 °C. However, in order to obtain the LST as accurately as possible, it is advisable to select coefficients according to the temperature range in the image.

The SWA is derived from a first-order Taylor-series linearization of the radiative transfer equation [21], and its formulation by Qin *et al.* [18] takes the general form of:

(3) $$T_{s}=A_{0}+A_{1}T_{10}-A_{2}T_{11}$$

where *T_s_* is the LST, *T_10_* and *T_11_*are the brightness temperatures of TIRS bands 10 and 11, respectively, and *A_0_*, *A_1_*, and *A_2_* are coefficients determined by the atmospheric transmittance and LSE in both TIRS bands:

(4a) $$A_{0}=E_{1}{\text{a}}_{10}+E_{2}a_{11}$$

(4b) $$A_{1}=1+A+E_{1}{\text{b}}_{10}$$

(4c) $$A_{2}=A+E_{2}b_{11}$$

Based on the algorithm developed by Qin *et al.* [18], we define:

(5) $$C_{i}=\varepsilon_{i}\tau_{i}\left(\theta \right)$$

(6) $$D_{i}=\left[1-\tau_{i}\left(\theta \right)\right]\left[1+\left(1-\varepsilon_{i}\right)\tau_{i}\left(\theta \right)\right]$$

where *ε_i_* is the LSE of band *i* and *τ_i_*(*θ*) is the atmospheric transmittance for a given zenith view angle *θ* in band *i*. Accordingly, the parameters in Equation (4) are defined:

(7) $$A=D_{10}/E_{0}$$

(8) $$E_{1}=D_{11}\left(1-C_{10}-D_{10}\right)/E_{0}$$

(9) $$E_{2}=D_{10}\left(1-C_{11}-D_{11}\right)/E_{0}$$

(10) $$E_{0}=D_{11}C_{10}-D_{10}C_{11}$$

The algorithm in Equation (4) uses the estimation of two geophysical parameters, namely atmospheric transmittance and LSE, in order to estimate the LST. The next section will briefly discuss the estimation of these parameters.

## 3. Estimating the Input Parameters of the SWA

### 3.1. Determination of Atmospheric Transmittance

Some SWAs were developed for wide swath sensors (e.g., NOAA-AVHRR, MODIS), and consequently emphasize the correction for zenith view angle effects on the atmospheric transmittance. However, in the case of the Landsat-8 TIRS at an altitude of 705 km with a swath of 185 km, the maximum zenith view angle is about 7.5°. At that angle, the effect on the atmospheric transmittance in both LWIR bands is negligible [18]. Thus, the term *θ* may be removed from Equations (5) and (6) for the purpose of implementing this SWA for TIRS.

In addition, transmittance is wavelength dependent and, therefore, different for each of the TIRS bands. Absorption in the 10.5–12.5 μm atmospheric window is mainly affected by water vapor that has a high spatial variability, since other atmospheric gases, such as *CO*_2_, *N*_2_, and *O*_3_, are well-mixed, and their effect can be considered constant throughout an image for the purpose of this analysis [22,23]. The atmospheric water vapor content, at the time of image acquisition, can be obtained from local measurements *in-situ*, or at nearby meteorological stations. The sun photometer measurements of the AErosol RObotic NETwork (AERONET), operated by NASA/GSFC [24], are suggested as a good source of water vapor data by Qin *et al.* [18], but they only provide data when a direct line of sight can be established between the station and the sun. Thus, sun photometer readings are a good source of information only for the processing of daytime and clear sky images.

Unlike LSE (discussed in the next section), it is not practical to estimate the atmospheric transmittance per pixel. Consequently, when using sun photometer point measurements, we make a latent assumption that the atmosphere is constant throughout the scene. While this is not always the case, this first-order estimation of the atmospheric transmittance for the whole scene, which relies on measured water vapor, is preferable to not accounting for it at all [25]. To roughly meet this assumption, cloud pixels must be masked. The atmospheric transmittance can then be simulated for the entire scene, based on the point measurement of the total content of water vapor in the column and standard atmospheric profiles using radiative transfer models, such as Modtran.

The use of sun photometers for estimating water vapor has some limitations. As mentioned earlier, they do not provide information when the sky is cloudy or at night. Furthermore, due to the lack of global coverage by ground-based instruments, AERONET data might not be available for all TIRS users for estimating the atmospheric water vapor content, especially in the northern latitudes, Asia, Australia, and central Africa. In these areas, other means are suggested, such as the use of a total column water vapor product provided by European Centre for Medium-Range Weather Forecasts (ECMWF) or the Moderate Resolution Imaging Spectroradiometer (MODIS) Precipitable Water product (MOD05_L2, MYD05_L2). While these products' spatial resolutions are significantly coarser than TIRS, they capture some spatial variability in water vapor distribution. Accordingly, ECMWF and MODIS data can be used when the users wish to refrain from assuming constant water vapor throughout the scene. The disadvantage of these data products is that in many cases, they do not accurately represent the state of the atmosphere at the time of TIRS overpass. Unlike the sun photometer that can perform measurements instantaneously or at a close temporal proximity to TIRS, the water vapor estimations derived from these products may be produced within several hours of the TIRS acquisition, and thus do not capture the exact atmospheric conditions at the acquisition time. This is indeed a limitation, and for this reason, these products should be used with caution. As a rule, users should select the data source according to its accessibility. When multiple sources of data are available, temporal proximity to the TIRS image acquisition and spatial proximity of the water vapor measurement site to the study area should be preferred. When facing a dilemma between temporal and spatial precision, the users have to consider this trade-off and make an optimal decision based on their experience.

The results of Modtran 4.0 simulations, conducted for a mid-latitude summer and for a 1976 standard US atmospheric profile to determine the relation between water vapor and atmospheric transmittance, are presented in Table 2. Throughout this paper, the model developed for mid-latitude summer is used as an example. Users may opt to use the coefficients for the 1976 standard US atmospheric profile where the atmosphere is modeled better. Based on AERONET measurements, the water vapor content in the current research area, in the northern Negev Desert, Israel, ranges from 0.5 to 3 g·cm^−2^. As can be seen in Table 2, the relation between water vapor and transmittance is close to linear. Qin *et al.* [18] showed that when this relation is evaluated for a larger range of water vapor values, it is better to divide the range into several sections and evaluate each of them separately in order to achieve a better accuracy. Since the atmosphere in our research area is relatively dry, and consequently, the range of water vapor values is relatively small, we are satisfied with the accuracy achieved by treating the entire range as a whole. Taking into account a plausible error in the water vapor content estimation of 0.2 g·cm^−2^, as suggested by Qin *et al.* (2001) [18] and according to the regression coefficients in Table 2, we can determine that the error in the atmospheric transmittance estimation is less than 0.031, which is slightly lower than the value obtained by Qin *et al.* [18].

### 3.2. Determination of LSE

The emissivity of land, in contrast to that of the ocean, is significantly different than unity, and varies with the heterogeneity of vegetation, surface moisture, roughness, and viewing angle [26]. Since LSE can change substantially over short distances, it is important to estimate its value for every pixel prior to applying the SWA. Several methods have been suggested to estimate the emissivity for other sensors and can also be applied to TIRS. Techniques for emissivity estimations from infrared and visible data are reviewed and discussed in detail elsewhere [27,28]. Adapting some of these techniques for use with the Landsat-8 requires the use of OLI bands to indirectly estimate the LSE in the TIRS bands. For instance, the LSE could be obtained from a land-cover classification, in which the emissivity values for each class are assumed [29]. This type of approach is exercised for MODIS LST and emissivity products. However, the estimated emissivity in arid and semi-arid areas is potentially uncertain, and users are advised to exercise caution in their applications. Of course, since vegetative cover tends to change with time [30], good knowledge of the study site and *in situ* LSE measurements of representative ground covers of the different classes that coincide with the satellite overpass are desired. However, if the required conditions for the classification approach are not met, it is possible to use NDVI by retrieving the proportions of soil and vegetation in order to estimate the LSE [31–33].

## 4. Sensitivity Analysis

Several scenarios were considered in order to estimate the possible LST estimation error due to misestimating the SWA input parameters: atmospheric transmittance and LSE. These scenarios included an LST range of 0–60 °C and a *T_10_*–*T_11_* range from −3 to 3 °C.

### 4.1. Sensitivity Analysis to Water Vapor Content

Since transmittance is derived from atmospheric water vapor content, it is expected that transmittance estimation errors will occur simultaneously in TIRS bands 10 and 11. Therefore, the sensitivity analysis was conducted for water vapor content, which serves as the input to the model, and by which the simultaneously occurring transmittance errors can be estimated according to the regression coefficients in Table 2.

**Figure 3.**
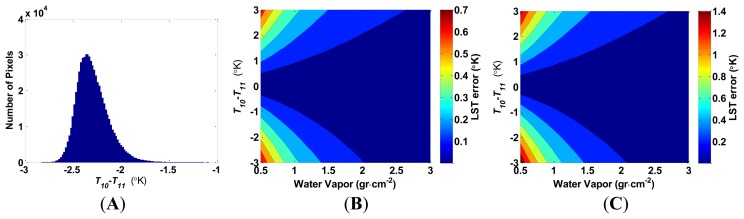
(**A**) Histogram of *T_10_*–*T_11_* computed from the Negev (Israel)—Sinai (Egypt) image acquired on 30 June 2013, path 41 row 206; (**B**) The increase in land surface temperature (LST) error (°K) as a result of underestimating the water vapor content in the atmospheric column by 0.1 g·cm^−2^, for different *T_10_*–*T_11_* scenarios, *e_10_* = 0.967, *e_11_* = 0.971; (**C**) The increase in LST error (°K) as a result of underestimating the water vapor content in the atmospheric column by 0.2 g·cm^−2^ under the same assumptions as in Figure 3B.

Estimation error of LST is almost independent of temperature change. It changes less than ±0.02 °C over the temperature range of 0–60 °C, assuming *T_10_*–*T_11_* = −2.3 (the average case for the Sinai-Negev dune field, as seen in Figure 3A), *e_10_* = 0.967, *e_11_* = 0.971, and underestimating the water vapor content in the atmospheric column by 0.2 g·cm^−2^ for the water vapor content range of 0.5–3 g·cm^−2^. This minute change is practically negligible.

**Figure 4.**
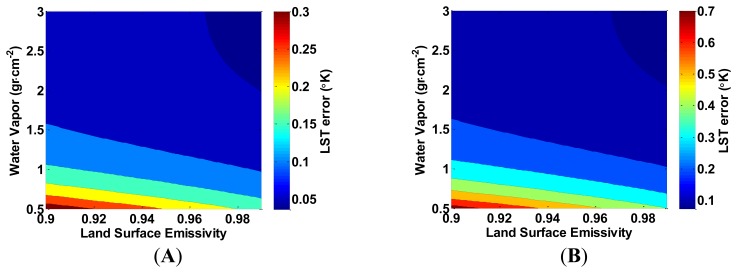
The increase in land surface temperature error (°K) as a result of underestimating the water vapor content in the atmospheric column by (**A**) 0.1 g·cm^−2^; and (**B**) 0.2 g·cm^−2^, as a function of the land surface emissivity. *T_10_*–*T_11_* is kept constant at 1 °K.

The LST estimation error increases when the atmospheric water vapor content decreases (and, thus, the atmospheric transmittance increases). This effect increases rapidly as the brightness temperature difference between TIRS bands 10 and 11 increases, as seen in Figure 3B,C. However, the contribution of the water vapor estimation error to the LST estimation is complex and also depends on the emissivity in both channels, as seen in Figure 4.

### 4.2. Sensitivity Analysis to LSE

**Figure 5.**
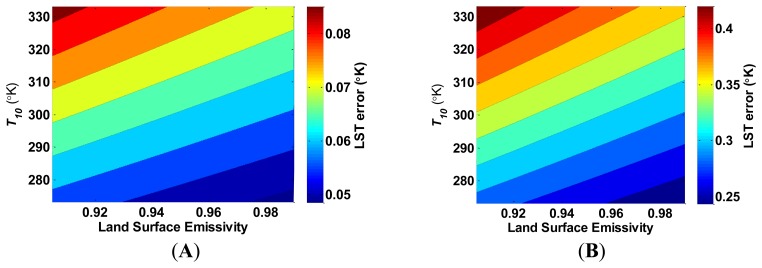
The increase in land surface temperature (LST) error (°K) as a result of underestimating the land surface emissivity (LSE) by (**A**) 0.001; and (**B**) 0.005, in both bands as a function of LSE and LST. *T_10_*–*T_11_* is kept constant at 1 °K, and the water vapor content is assumed to be 1.5 g·cm^−2^.

An error in LSE estimation can occur simultaneously for both of the TIRS bands, but a separate error for each of the bands is possible. In the following analysis, we present the example of a simultaneous error in both bands. Unlike the relative indifference of the LST estimation error to temperature when the water vapor content is misestimated, the LST error is sensitive to temperature when the LSE is misestimated. Figure 5A,B presents this sensitivity.

**Figure 6.**
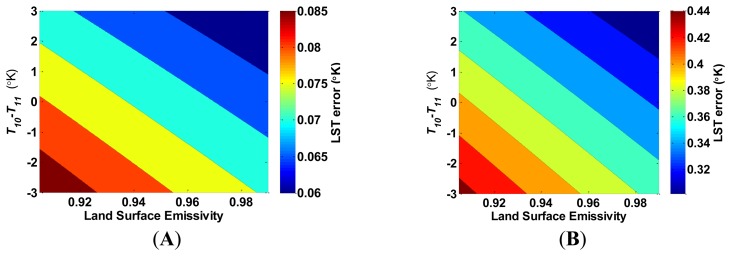
The increase in land surface temperature error (°K) as a result of underestimating the land surface emissivity (LSE) by (**A**) 0.001; and (**B**) 0.005, in both bands as a function of LSE and *T_10_*–*T_11_*. The water vapor content is assumed to be 1.5 g·cm^−2^ and *T_10_* = 313.15 °K.

**Figure 7.**
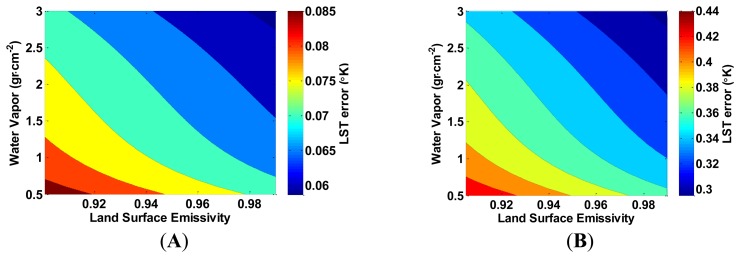
The increase in land surface temperature error (°K) as a result of underestimating the land surface emissivity (LSE) by (**A**) 0.001; and (**B**) 0.005, in both bands as a function of LSE and water vapor content. *T_10_*–*T_11_* is kept constant at 1 °K, and *T_10_* = 313.15 °K.

The LST error increases linearly, with the LST, and decreases linearly when the LSE increases. Figure 6A,B presents a similar linear dependence of the LST error by *T_10_*–*T_11_*; as *T_10_*–*T_11_* increases, the LST error decreases. In both Figures 5 and 6, the change of the LST error increases at the same rate as the LSE estimation error. In addition, the LST estimation error is sensitive to the water vapor content when the LSE is misestimated, as depicted in Figure 7. When water vapor content is higher, and the atmospheric transmittance is lower, the LST error decreases. Figures 4, 5, 6 and 7 show that the LST error is oppositely related to the LSE.

## 5. Accuracy Assessment of the Proposed SWA

In order to assess the accuracy of our SWA, we used Modtran 4.0 to simulate the thermal radiance reaching the sensor for an input Mid-Latitude Summer atmospheric profile with known LST and LSE. The simulated radiance was then converted into brightness temperatures for the TIRS bands, and used as inputs to the SWA. The LST estimation errors for 60 different scenarios, featuring different combinations of LST, LSE, and atmospheric water vapor content, are presented in Table 3. The RMSE for the LST estimation errors in Table 3 is 0.93 °C.

## 6. Summary

We presented a SWA for Landsat-8 TIRS data. The ability to derive LST temperatures accurately has immediate impacts for potential Landsat-8 users and applications. As data from the new Landsat-8 are distributed freely, its high resolution thermal abilities can allow for new scientific advances in earth science. Although the spatial resolution of TIRS is degraded to 100 m, in comparison with the 60 m resolution of the Landsat-7 ETM+ thermal band, the added value of having two bands is more accurate LST estimations with TIRS than with its predecessor. In addition, the 100 m resolution is sufficient for water consumption measurements over fields irrigated by center pivot systems [9], as well as for other uses over relatively homogeneous areas.

In this paper, we have outlined possible strategies for evaluating the atmospheric water vapor content and LSE. When selecting a data source for estimating the water vapor content, the tradeoff between temporal and spatial precision has to be considered by the users on a case-by-case basis. Strategies to estimate LSE, based on techniques employed by previous sensors, have been suggested. These techniques can still be refined and adapted for OLI's new spectral band configuration, which is different than its predecessors. Therefore, future work should focus on evaluating the best methods of estimating the SWA input parameters (e.g., atmospheric transmittance, LSE), the cross-sensor validation of the input parameters and the resulting LST.

## Figures and Tables

**Table 1.** Regression coefficients (*a*, *b*), coefficient of determination (*r^2^*), and Standard Error of Estimate (SEE) of the derivative of the Planck function, ∂*B*, for Landsat-8 TIRS bands 10 and 11, for estimating parameter *L_i_* at different ranges of temperatures (*T*).

*_T_***Range (°C)**	***a****_10_*	***b****_10_*	$r_{10}^{2}$	***SEE****_10_*	***a****_11_*	***b****_11_*	$r_{11}^{2}$	***SEE****_11_*
0–30	−59.1391	0.4213	0.9991	0.0424	−63.3921	0.4565	0.9991	0.0438
0–40	−60.9196	0.4276	0.9985	0.0746	−65.2240	0.4629	0.9985	0.0769
10–40	−62.8065	0.4338	0.9992	0.0415	−67.1728	0.4694	0.9992	0.0427
10–50	−64.6081	0.4399	0.9986	0.0730	−69.0215	0.4756	0.9986	0.0750

**Table 2.** Relationship between atmospheric transmittance and water vapor content in the column for the water vapor content range of 0.5–3 g·cm^−2^.

**Profile**	**Estimation Equation**	*_r_*^2^	*_SEE_*
1976US Standard	*τ*_10__=_ −0.1146*w* + 1.0286	0.9882	0.0094
	*τ*_11__=_ −0.1568*w* + 1.0083	0.9947	0.0086

Mid-Latitude Summer	*τ*_10__=_ −0.1134*w* + 1.0335	0.986	0.0101
	*τ*_11__=_ −0.1546*w* + 1.0078	0.996	0.0073

**Table 3.** Estimation errors of Land Surface Temperature (LST) for various simulated combinations of LST, Land Surface Emissivity (LSE), and atmospheric water vapor content.

**Water Vapor (g·cm^−2^)**	**LST (°C)**	**LSE = 0.98**	**LSE = 0.97**	**LSE = 0.96**	**LSE = 0.95**
1	10	0.1456	0.6979	1.2502	1.8249
	20	0.0959	0.6885	1.2912	1.8939
	30	−0.0021	0.6410	1.2840	1.9372
	40	−0.0933	0.5777	1.2712	1.9647
	50	−0.2249	0.4966	1.2304	1.9742

2	10	−0.3264	0.2512	0.7819	1.3411
	20	−0.5057	0.0937	0.6932	1.2927
	30	−0.7877	−0.1479	0.4919	1.1418
	40	−1.1519	−0.4902	0.1999	0.9086
	50	−1.5968	−0.8663	−0.1073	0.6232

3	10	−1.1184	−0.6097	−0.0698	0.4701
	20	−0.8608	−0.2806	0.3208	0.9010
	30	−0.8268	−0.1852	0.4565	1.0770
	40	−0.9127	−0.2308	0.4512	1.1331
	50	−1.1507	−0.4073	0.3250	1.0472

## Notes

**Author Contributions:** Offer Rozenstein was principal to all phases of the investigation, including the atmospheric modeling, analysis, simulation of the algorithm, the literature review, and manuscript preparation. Zhihao Qin contributed to the algorithm adaptation and interpretation of the modeling results. Yevgeny Derimian contributed to the algorithm adaptation, the atmospheric modeling, and interpretation of the modeling results. Arnon Karnieli conceived the idea for the project, and supervised and coordinated the research activity. All authors participated in the editing of the paper.
