# Correction: Rozenstein, O., *et al.* Derivation of Land Surface Temperature for Landsat-8 TIRS Using a Split Window Algorithm. *Sensors* 2014, *14*, 5768–5780

**DOI:** 10.3390/s140611277

**Published:** 2014-06-24

**Authors:** Offer Rozenstein, Zhihao Qin, Yevgeny Derimian, Arnon Karnieli

**Affiliations:** 1 The Remote Sensing Laboratory, Jacob Blaustein Institutes for Desert Research, Ben-Gurion University of the Negev, Sede Boker Campus, Midreshet Ben-Gurion 84990, Israel; E-Mail: oferroz@yahoo.com; 2 Institute of Agricultural Resources and Regional Planning, Chinese Academy of Agricultural Sciences, Beijing 100081, China; E-Mail: qinzh@caas.net.cn; 3 Laboratoire d'Optique Atmosphérique, Université de Lille1/CNRS, Villeneuve d'Ascq 59655, France; E-Mail: Yevgeny.Derimian@univ-lille1.fr

We have recently been made aware by a reader of a typo in Equation (4a) of our recent paper [1]. The equation currently reads as follows:

(4a) $$A_{0}=E_{1}a_{10}+E_{2}a_{11}$$

We would like to make the following corrections:

(4a) $$A_{0}=E_{1}a_{10}-E_{2}a_{11}$$

This sign correction does not affect the modeling work presented in the paper, since the mistake appears only in the text, and the correct sign was used in the model. The authors would like to apologize for any inconvenience caused to the readers by these changes.
